# Maternity Waiting Home Interventions as a Strategy for Improving Birth Outcomes: A Scoping Review and Meta-Analysis

**DOI:** 10.5334/aogh.3496

**Published:** 2022-01-18

**Authors:** Samantha Smith, Hannah Henrikson, Rita Thapa, Suresh Tamang, Ruma Rajbhandari

**Affiliations:** 1 Brigham and Women’s Hospital, Department of Medicine, Division of Global Health Equity, Boston, Massachusetts, USA; 2 Nick Simons Institute, Sanepa, Lalitpur, Nepal; 3 Harvard Medical School, Boston, Massachusetts, USA; 4 Mount Auburn Hospital, Department of Medicine, Division of Gastroenterology, Cambridge, Massachusetts, USA

## Abstract

**Background and Objective:**

Over 300 000 women worldwide die due to pregnancy-related complications annually, with most occurring in developing countries where access to skilled obstetric care is limited. Maternity waiting homes (MWHs) are one intervention designed to increase access to skilled prenatal care in resource-limited settings. MWHs are defined as accommodations at or near a health facility where pregnant women can stay in the final weeks of their pregnancy so they can be easily transferred to the health facility to give birth. While MWHs have existed for decades, evidence regarding their effectiveness in reducing adverse birth outcomes has been mixed. The objective of this study is to comprehensively assess all available MWH research reporting quantitative maternal and childbirth data to determine whether MWHs are an effective maternal health strategy in resource-limited settings.

**Methodology:**

We conducted a scoping review and meta-analysis of existing literature on MWHs according to PRISMA guidelines. Descriptive statistics and odds ratios were calculated for the following birth outcomes: maternal mortality, perinatal mortality, and caesarian section. Quantitative analysis was conducted in RStudio and Stata Version 16.

**Results:**

One hundred seventy-one records were retrieved from our initial database search, of which 66 were identified as relevant. Only 15 of these records reported quantitative data on the health outcomes of interest and therefore met inclusion criteria for our meta-analysis. All studies reporting maternal mortality demonstrated a protective effect of MWHs (aggregate OR: 0.19 [0.10, 0.40]), as did all studies reporting perinatal mortality (aggregate OR: 0.29 [0.16, 0.53]). Studies reporting caesarian section were more varied and indicated less of a protective effect (aggregate OR: 1.80 [1.18, 2.75]).

**Conclusions:**

There is some indication that MWHs are an effective strategy for reducing maternal and perinatal mortality in resource-limited settings. However, our analysis was constrained by the observational design of most prior MWH studies. More rigorous MWH evaluations, ideally in the form of randomized-control trials, are needed to better determine MWH effectiveness.

## Introduction

Over 300 000 women die due to pregnancy-related complications every year, and 99% of these deaths occur in developing countries [1]. The vast majority of maternal deaths are preventable and treatable when women have access to skilled obstetric care, but this is challenging in resource-limited settings. A widely accepted framework for understanding maternal mortality is the three phases of delay model, which describes three main periods where women are likely to die in pregnancy: 1) delay in deciding to seek medical care, 2) delay in reaching a care facility, and 3) delay in receiving care once arriving at a facility [2]. Maternity waiting homes (MWHs) are one intervention aimed at eliminating the phase 2 delay by bringing women closer to facilities for delivery. MWHs go by many names (e.g., maternity waiting areas, antenatal villages), but in general are accommodations at or near a health facility where pregnant women can stay in the final weeks of their pregnancy so they can easily be transferred to the health facility to give birth with a skilled attendant present and with emergency obstetric care available if needed [3].

Although MWHs have existed for decades, literature regarding the effectiveness of MWHs on improving maternal and child health outcomes has been mixed. Several researchers have attempted literature reviews on the topic of MWHs, which are either too narrow in their eligibility criteria or are not comprehensive. In 1996, the World Health Organization (WHO) Safe Motherhood Unit conducted a review of experiences as case studies of MWHs in seven countries [4]. Most studies in this review demonstrated improved health outcomes, but challenges related to record-keeping and data collection in resource-limited settings proved obstructive to quantitative analysis. This made it challenging for the review to assess MWH effectiveness beyond anecdotal evidence. Even so, the WHO review created a framework for implementing MWH interventions with four essential elements and seven steps for establishing new MWHs. Overall, the WHO Safe Motherhood Unit recommended establishing MWHs as part of a comprehensive program to prevent pregnancy-related morbidity and mortality [4].

In an attempt to conduct a more rigorous review, researchers from the Netherlands published a Cochrane review of MWH randomized control trials (RCTs), finding that no RCTs or cluster RCTs had been conducted as of 2012 [5]. The review instead included 9 retrospective cohort studies, but was restricted in its interpretation due to high risk of selection bias. In contrast to the aforementioned WHO review, almost half of the studies included in the Cochrane review found MWHs to be ineffective in improving health outcomes. The authors called on future studies to assess MWH effectiveness using RCTs rather than observational studies.

More recently, two reviews focused on MWH interventions were published in 2017. The first, a scoping review in *Maternal and Child Health Journal*, focused specifically on newborn outcomes [6]. The authors found that in general, MWHs have positive impacts on newborn morbidity and mortality, but highlighted the need for unbiased research into the effectiveness of MWHs [6]. It should be noted that this review was scoping and not comprehensive, and was limited by excluding maternal health outcomes from analysis [6]. The second MWH review, published in *BMC Pregnancy and Childbirth*, included 29 studies and focused on qualitative findings of MWH research [7]. While this review did not analyze quantitative data, it provided important insight into the implementation of MWH interventions. The authors emphasized the importance of engaging women and other community members for input and to identify barriers, the quality of MWH facilities, the quality of maternity care provided at the associated health facility, and the financial and operational stability of MWHs [7].

The objective of this literature review and meta-analysis is to comprehensively assess all available research reporting quantitative MWH maternal and childbirth outcome data, aggregate and analyze health outcome measures across studies, and determine whether MWHs are effective at improving maternal and child health outcomes in resource-limited settings.

## Methods

### Search Strategy and Selection Criteria

This scoping review and meta-analysis was conducted in accordance with PRISMA guidelines [8]. The review protocol was registered in PROSPERO (number CRD42018111716). We searched the electronic bibliographic databases PubMed and EMBASE. The search strategy included only terms relating to the intervention. The following terms were searched: “Maternity waiting home,” “maternal waiting home,” “maternity waiting area,” “maternity waiting facility,” “maternity waiting shelters,” and “antenatal village.” Studies were restricted to those that describe an intervention in a low- or middle-income country. There were no language or date restrictions. Studies were excluded if they were literature reviews, not focused on an MWH intervention, or did not report hard outcome data. Database searches were re-run prior to final analysis and further studies were retrieved for inclusion.

### Data Synthesis and Analysis

A narrative synthesis of both quantitative and qualitative findings from included studies was produced. This synthesis was structured around study design, study population, key findings and implications, and characteristics of the MWH intervention (e.g., cost, admission criteria, facilities, etc.). Quantitative data synthesis was performed by calculating descriptive statistics and odds ratios for the following outcomes: maternal mortality, perinatal mortality/stillbirth, and caesarian section. Maternal mortality is defined as the number of maternal deaths divided by the total number of births per study. Perinatal mortality is defined as the number of perinatal deaths divided by the total number of births per study. Stillbirth, which we used as a proxy for perinatal mortality in studies that do not report perinatal deaths, is defined as the number of stillbirths or intrauterine fetal deaths divided by the total number of births per study. Caesarian section rate is defined as the total number of C-sections divided by the total number of births. Initial quantitative data analysis was performed using RStudio and finalized using Stata Version 16 (StataCorp, College Station, TX). All meta-analysis functions were performed in Stata Version 16.

## Results

**Figure 1:**
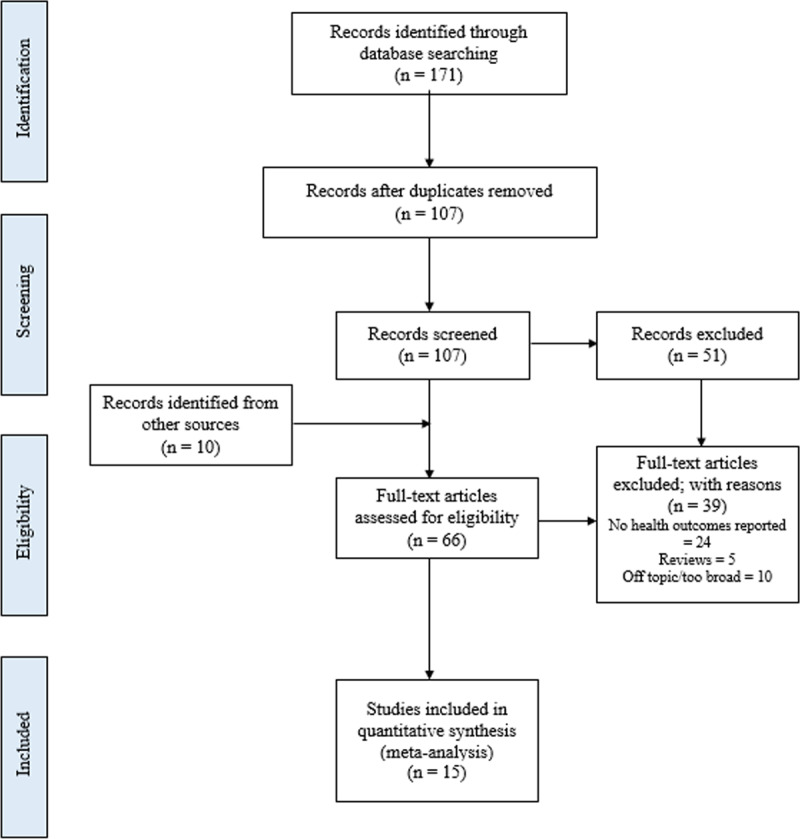
PRISMA Diagram demonstrating search strategy and inclusion criteria for the scoping review and meta-analysis.

Our database search yielded 171 records, with 107 remaining after the removal of duplicates. In addition, 10 records were identified from other sources, such as the references of prior reviews. Twelve records were excluded because they were not full-text, 5 articles were excluded because they are reviews, 10 articles were excluded for being off-topic or otherwise too broad, and 24 articles were excluded for lacking health outcome data. This left 66 total articles: 34 articles with quantitative outcome data and 32 articles with qualitative health outcome data (***Figure 1***). After a rigorous review of these studies, we identified only 15 articles reporting health outcomes of interest: maternal mortality, perinatal mortality or stillbirth, and caesarian section. These 15 studies were selected for inclusion in the final meta-analysis.

All 66 records retrieved were observational. There was great variance in terms of study design, with cohort studies [9,10,11,12,13,14,15,16,17,18], cross-sectional studies [19,20,21,22,23,24,25,26,27,28,29,30,31,32,33,34,35,36,37,38,39], intervention studies [40,41,42,43,44,45,46,47,48,49,50], and unspecified studies included [51,52,53,54,55,56,57,58,59,60,61,62,63,64,65,66,67,68,69,70,71,72,73,74,75]. Of the 66 studies, only one mentioned any kind costing analysis of MWHs [47]. Although minimal restrictions were placed on MWH location, each of the 15 studies included in the meta-analysis took place in sub-Saharan Africa (Appendix 1). Among the original 66 studies, most took place in sub-Saharan Africa, Latin America, and Southeast Asia.

**Figure 2:**
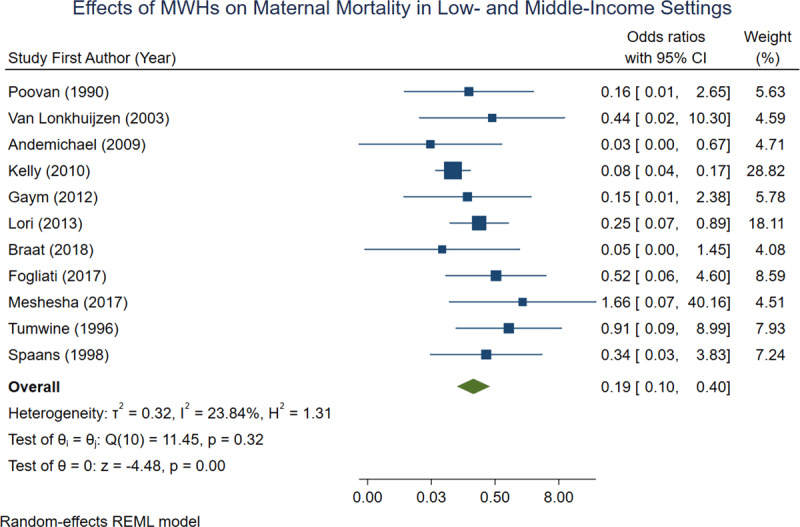
Forest plot demonstrating effects of MWHs on maternal mortality across 11 observational studies.

Of the 15 studies included in the meta-analysis, 11 reported maternal mortality data (***Table 1***). Each of these 11 studies demonstrated a lower prevalence of maternal mortality in women who utilized an MWH compared to those who did not (***Table 1***). Six of the studies reported no maternal deaths in the group of women who utilized an MWH (***Table 1***). When aggregated, 0.05% of mothers who utilized an MWH died of pregnancy-related complications, compared to 0.6% of mothers who did not utilize an MWH. In a random-effects REML model, the overall odds ratio for maternal mortality was 0.19 [0.10, 0.40] (***Figure 2***).

**Table 1:** Maternal mortality analysis comparing women who stayed in an MWH prior to giving birth to women who did not stay in an MWH prior to giving birth across 11 observational studies. n = number of women enrolled in study; MM = maternal mortality, OR = odds ratio.


FIRST AUTHOR	TOTAL N	MWH N	NON-MWH N	MM MWH	PERCENT MATERNAL DEATH	MM NON-MWH	PERCENT MATERNAL DEATH	ADJUSTED OR

Poovan	777	142	635	0	0.00%	13	2.04%	0.16

Van Lonkhuijzen	510	218	292	0	0.00%	1	0.34%	0.44

Andemichael	1128	862	266	0	0.00%	5	1.88%	0.03

Kelly	24148	6805	17343	6	0.08%	187	1.08%	0.08

Gaym	4275	902	3373	0	0.00%	12	0.36%	0.15

Lori	18044	8477	9567	3	0.03%	12	0.13%	0.25

Braat	17679	2784	14895	0	0.00%	51	0.34%	0.05

Fogliati	1077	348	729	1	0.29%	4	0.55%	0.52

Meshesha	516	86	430	0	0.0%	1	0.23%	1.66

Tumwine	1053	280	773	1	0.36%	3	0.39%	0.91

Spaans	1041	616	425	1	0.16%	2	0.47%	0.34

**Total**	70248	21520	48728	12	0.05%	291	0.60%	0.19


**Figure 3:**
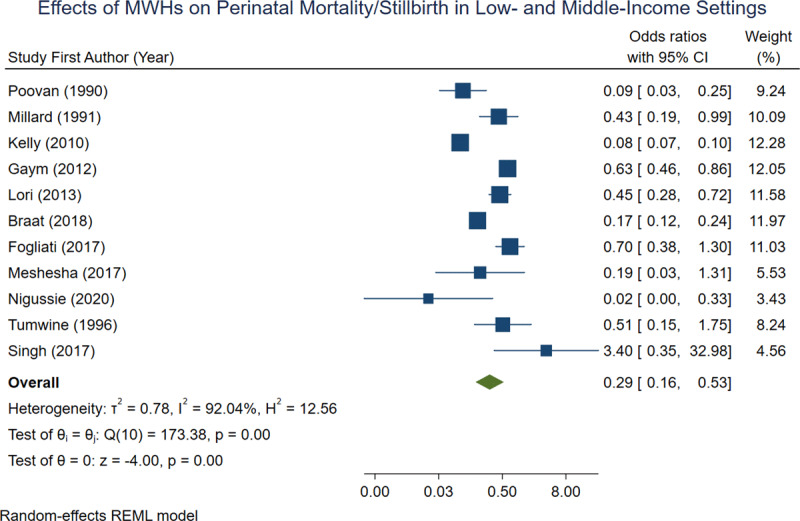
Forest plot demonstrating effects of MWHs on perinatal mortality or stillbirth across 11 observational studies.

Of the studies included in the meta-analysis, 11 reported perinatal mortality or stillbirth (***Table 2***). Across all included studies, there was a lower prevalence of perinatal mortality or stillbirth in women who utilized an MWH compared to those who did not (***Table 2***). When aggregated, 1.30% of MWH births resulted in perinatal death, compared to 10.42% of non-MWH births. In a random-effects REML model, the overall odds ratio for perinatal mortality/stillbirth was 0.29 [0.16, 0.53] (***Figure 3***).

**Table 2:** Perinatal mortality analysis comparing women who stayed in an MWH prior to giving birth to women who did not stay in an MWH prior to giving birth across 11 observational studies.


FIRST AUTHOR	TOTAL N	MWH N	NON-MWH N	PM MWH	PERCENT PERINATAL DEATH	PM NON-MWH	PERCENT PERINATAL DEATH	ADJUSTED OR

Poovan*	777	142	635	4	2.82%	161	25.35%	0.09

Millard	854	502	352	17	3.39%	24	6.82%	0.43

Kelly*	24148	6805	17343	120	1.76%	3316	19.12%	0.08

Gaym*	4275	902	3373	48	5.32%	276	8.18%	0.63

Lori	18044	8477	9567	24	0.28%	60	0.63%	0.45

Braat*	17679	2784	14895	38	1.36%	1110	7.45%	0.17

Fogliati	1077	348	729	14	4.02%	41	5.62%	0.70

Meshesha*	516	86	430	1	1.16%	25	5.81%	0.19

Nigussie	829	323	506	0	0.00%	43	8.50%	0.02

Tumwine	1053	280	773	3	1.07%	16	2.07%	0.51

Singh	141	46	95	0	0.00%	4	4.21%	3.40

**Total**	69393	20695	48698	269	1.30%	5076	10.42%	0.29


n = number of women enrolled in study; PM = perinatal mortality, OR = odds ratio; * Stillbirth used as proxy for perinatal mortality.

**Figure 4:**
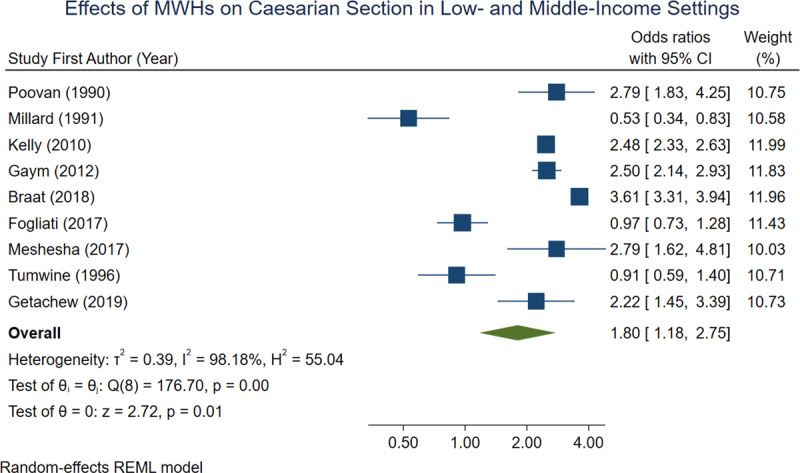
Forest plot demonstrating effects of MWHs on C-section across 9 observational studies.

Nine of the 15 studies reported number of caesarian sections, 5 of which showed a higher rate of C-sections among MWH users compared to non-users (***Table 3***). When aggregated 36.2% of women who stayed in an MWH had a C-section, compared to 18.4% of those who did not stay in an MWH. In a random-effects REML model, the overall odds ratio for caesarian section was 1.80 [1.18, 2.75] (***Figure 4***).

**Table 3:** Caesarian section analysis comparing women who stayed in an MWH prior to giving birth to women who did not stay in an MWH prior to giving birth across 9 observational studies. n = number of women enrolled in study; OR = odds ratio.


FIRST AUTHOR	TOTAL N	MWH N	NON-MWH N	C-SECTION MWH	PERCENT C-SECTION	C-SECTION NON-MWH	PERCENT C-SECTION	ADJUSTED OR

Poovan	777	142	635	44	31.00%	88	13.86%	2.79

Millard	854	502	352	37	7.37%	46	13.07%	0.53

Kelly	24148	6805	17343	2632	38.68%	3520	20.30%	2.48

Gaym	4275	902	3373	347	38.47%	674	19.98%	2.50

Braat	17679	2784	14895	1145	41.13%	2414	16.21%	3.61

Fogliati	1077	348	729	104	29.89%	222	30.45%	0.97

Meshesha	516	86	430	25	29.07%	55	12.79%	2.79

Tumwine	1053	280	773	32	11.43%	96	12.42%	0.91

Getachew	812	406	406	72	17.73%	36	8.87%	2.22

**Total**	51191	12255	38936	4438	36.21%	7151	18.37%	1.80


Of the original 66 articles reviewed, all reported at least some descriptive information about MWHs. These qualitative data included information about the direct cost of staying at an MWH, the admission criteria for an MWH, and whether or not food is provided to women at an MWH (Appendix 2). Twenty-eight studies reported whether there was a direct cost to MWH users, 13 of which did charge women a fee, and 15 of which were free of charge (***Table 4***). Twenty-one studies reported whether or not there were admission criteria for staying in the MWH, 11 of which specified admission criteria and 10 of which reported no criteria (***Table 4***). Forty-three studies described the provisions or facilities offered by MWHs. Of these studies, 10 reported the provision of food, 18 reported the presence of toilet and shower facilities, and 15 reported the presence of kitchen facilities (***Table 4***). Very few studies provided information on how MWHs were owned and operated. Of the studies included in the meta-analysis, three reported the involvement of faith-based organizations in MWH operations, three reported support from their local ministries of health, four reported a community-ownership model supported by external development partners, and the remainder provided no operations information (Appendix 2). Based on these limited results, it appears that most MWHs are operated through either public support or public-private partnerships.

**Table 4:** Qualitative characteristics of MWHs identified from the 66 observational studies reviewed.


COUNTRY	ARTICLES RETRIEVED	DIRECT COST		ADMISSION CRITERIA			PROVISIONS AND FACILITIES		
		
		FEE CHARGED	FREE	HIGH RISK	FACILITY REFERRAL	NONE	FOOD PROVIDED	TOILET AND SHOWERS	KITCHEN FACILITIES

Ethiopia	14	1	7	3	–	1	5	3	3

Ghana	2	1	–	1	–	–	–	1	1

Guatemala	1	1	–	–	–	–	–	1	1

Kenya	1	–	–	–	–	–	–	1	1

Lao People’s Democratic Republic	1	–	–	–	–	–	–	1	1

Liberia	7	–	4	2	–	2	–	2	2

Malawi	5	–	–	–	–	2	2	3	2

Nicaragua	1	1	–	–	–	1	–	–	–

Nigeria	1	–	–	–	–	–	–	–	–

Tanzania	3	3	–	1	1	1	–	1	1

Timor-Leste	2	–	–	–	–	1	–	–	–

Zambia	19	4	–	1	–	–	1	4	3

Zimbabwe	9	2	4	1	1	2	2	1	–

Total	66	13	15	9	2	10	10	18	15


## Discussion

The results of this meta-analysis indicate that, among the included studies, MWHs are very effective at reducing both maternal mortality and perinatal mortality. This is especially true if MWHs are included as part of a larger, comprehensive maternal health strategy. The overall odds ratios calculated for maternal mortality and perinatal mortality are 0.19 and 0.29 respectively, indicating a strong protective effect of MWHs on birth outcomes. The overall odds ratio of 1.80 for caesarian section was less conclusive, however this could indicate that women who stay in MWHs have better access to emergency obstetric care when pregnancy complications arise. This is in contrast to women admitted directly to the hospital from home, who may be too far along in their labor to undergo C-section upon arrival.

These studies identified several factors that influenced women’s decisions to utilize MWHs. Direct cost and the provision of food were identified as major factors in deciding whether or not to stay at an MWH. Eligibility criteria also played an important, albeit controversial, role in MWH utilization. The studies included in this meta-analysis were split on the presence of specific admission criteria. In studies reporting admission criteria, commonly cited criteria included current or prior pregnancy complication and substantial distance from a health facility [9,12,13,14,15,43,55]. Other studies, however, reported that MWHs were open to all women approaching the end of their pregnancy, regardless of obstetric history or distance from a health facility [10,11,19,20,41,42,51,54]. While risk-based admission criteria for MWHs make sense, especially if the demand for utilization is high, it should be noted that 15–20% of “low-risk” pregnancies still result in emergency complications [76]. Therefore, the creation of MWH admission criteria based solely on observed risk could prohibit utilization by other women who stand to benefit from MWH services.

## Limitations

A major limitation of this scoping review and meta-analysis is that all of the studies included are observational, leaving them prone to bias. In addition, all of the studies use women who are directly admitted to a hospital for delivery as a comparison group, rather than women who give birth at home. This distinction is important as most rural women in low- and middle- income countries deliver at home. The use of women directly admitted to a hospital as a comparison group has potential to bias the results by overestimating the effect of MWHs on birth outcomes. This is due to that fact that women directly admitted to these hospitals often attempt to give birth at home first, seeking medical care only when complications arise. This makes the comparison group naturally more prone to adverse health outcomes. In future MWH evaluation studies, women who give birth at home may be a more valid comparison group. In addition, C-section may not be an ideal measure of MWH impact for future studies, given that patients requiring C-section are more likely to be accommodated at MWHs and therefore prone to sample selection bias.

Another limitation of this review and meta-analysis is the fact that limited research on MWHs exists. As noted, all 15 of the included studies were observational. Only one randomized control trial has been conducted to determine the efficacy of MWHs on improving birth outcomes [77]. This RCT was ultimately excluded from our meta-analysis because it only reported on the presence or absence of institutional births, and not on specific birth outcomes. Furthermore, only one economic evaluation has been conducted to determine the financial practicality of MWHs [47]. While this costing analysis did find MWHs to be a cost-effective intervention, the study was conducted in rural Liberia and therefore may not be generalizable to other locations [47]. Robust evaluation studies, both impact and economic, are needed to accurately assess the benefits of MWHs on reducing adverse birth outcomes.

## Conclusion

Overall, there is evidence to suggest that MWH interventions are protective against maternal mortality and perinatal mortality. Although current research on MWH effectiveness is observational and prone to bias, the aggregate effect measures of MWHs on maternal mortality and perinatal mortality are notable. MWH interventions warrant rigorous, unbiased evaluations in the form of RCTs or cluster RCTs. Ideally, future research will cluster-randomize facilities to establish an MWH and match facilities without an MWH as a comparison group. While this would take extensive resources and community support, it would allow researchers to adequately assess MWH effectiveness in low- and middle-income settings. Based on the results of this meta-analysis, low- and middle-income settings should strongly consider including MWHs as part of a comprehensive plan to improve maternal and child health.

## Additional Files

The additional files for this article can be found as follows:

10.5334/aogh.3496.s1 Appendix 1. TMWH Literature Review Summary Table.

10.5334/aogh.3496.s2 Appendix 2. MWH Selected Qualitative Characteristics.

## References

[1] World Health Organization. Maternal mortality. February 18, 2016. http://www.who.int/news-room/fact-sheets/detail/maternal-mortality

[2] Thaddeus S, Maine D. Too far to walk: Maternal mortality in context. Soc Sci Med. 1994; 38(8): 1091–1110. https://pdfs.semanticscholar.org/b417/80532a0b5d28547b1f659e64dacf36eac98d.pdf. DOI: 10.1016/0277-9536(94)90226-7 8042057

[3] Maternity Waiting Homes Alliance. Maternity waiting homes: How do they save lives? [Internet]. 2019. https://www.maternitywaitinghomes.org/maternity-waiting-homes.

[4] WHO Safe Motherhood Unit. Maternity waiting homes: A review of experiences. WHO/RHT/MSM. 1996: 96(21): 1–38. https://www.who.int.

[5] Van Lonkhuijzen L, Stekelenburg J, van Roosmalen J. Maternity waiting facilities for improving maternal and neonatal outcomes in low-resource countries. Cochrane Database Syst Rev. October 17, 2012; 10. https://www.cochrane.org/CD006759/PREG_maternity-waiting-facilities-for-improving-maternal-and-neonatal-outcomes-in-low-resource-countries. DOI: 10.1002/14651858.CD006759.pub3 PMC4098659 23076927

[6] Buser JM, Lori JR. Newborn outcomes and maternity waiting homes in low- and middle-income countries: A scoping review. Matern Child Health J. 2017; 21: 760–769. https://www.ncbi.nlm.nih.gov/pubmed/27475822. DOI: 10.1007/s10995-016-2162-2 27475822

[7] Penn-Kekana L, Pereira S, Hussein J, et al. Understanding the implementation of maternity waiting homes in low- and middle- income countries: A qualitative thematic synthesis. BMC Pregnancy Childbirth. 2017; 17: 269–281. DOI: 10.1186/s12884-017-1444-z 28854880 PMC5577673

[8] Moher D, Liberati A, Tetzlaff J, Altman DG, The PRISMA group. Preferred reporting items for systematic reviews and meta-analyses: The PRISMA statement. PLoS Med. July 21, 2009; 6(7): e1000097. DOI: 10.1371/journal.pmed.1000097 19621072 PMC2707599

[9] Poovan P, Kifle F, Kwast B. A maternity waiting home reduces obstetric catastrophes. World Health Forum. 1990; 11: 440–446. http://www.who.int/iris/handle/10665/45561. 2092704

[10] Millard P, Bailey J, Hanson J. Antenatal village stay and pregnancy outcome in rural Zimbabwe. Cent Afr Med J. 1991; 37(1): 1–4. https://www.ncbi.nlm.nih.gov/pubmed/2060001. 2060001

[11] Lori JR, Munro ML, Rominski S, et al. Maternity waiting homes and traditional midwives in rural Liberia. Int J Gynaecol Obstet. 2013; 123(2): 114–118. https://www.ncbi.nlm.nih.gov/pmc/articles/PMC3795996/. DOI: 10.1016/j.ijgo.2013.05.024 23992657 PMC3795996

[12] Kelly J, Kohls E, Poovan P, et al. The role of a maternity waiting area (MWA) in reducing maternal mortality and stillbirths in high-risk women in rural Ethiopia. BJOJ. 2010; 117: 1377–83. https://www.ncbi.nlm.nih.gov/pubmed/20670302. DOI: 10.1111/j.1471-0528.2010.02669.x 20670302

[13] Gaym A, Pearson L, Khynn Win WS. Maternity waiting homes in Ethiopia – three decades experience. Ethiop Med J. 2012; 50(3): 209–219. https://www.ncbi.nlm.nih.gov/pubmed/23409404. 23409404

[14] Braat F, Vermeiden T, Getnet G, Schiffer R, van den Akker T, Stekelenburg J. Comparison of pregnancy outcomes between maternity waiting home users and non-users at hospitals with and without a maternity waiting home: Retrospective cohort study. Int Health. January 1, 2018; 10(1): 47–53. https://www.ncbi.nlm.nih.gov/pubmed/29342256. DOI: 10.1093/inthealth/ihx056 29342256

[15] Van Lonkhuijzen L, Stegeman M, Nyirongo R, van Roosmalen J. Use of maternity waiting home in rural Zambia. Afr J Reprod Health. 2003; 7(1): 32–36. DOI: 10.2307/3583343 12828140

[16] JE Perosky, McLean KZ, Kofa A, Nyanplu A, Munro-Kramer ML, Lori JR. Utilization of maternity waiting homes: before, during and after the Ebola virus disease outbreak in Bong County, Liberia. International Health. 2020; 12(1): 69–71. https://academic.oup.com/inthealth/article/12/1/69/5531295?login=true. DOI: 10.1093/inthealth/ihz039 31294786 PMC6964226

[17] Chandramohan D, Cutts F, Millard P. The effect of stay in a maternity waiting home on perinatal mortality in rural Zimbabwe. J Trop Med Hyg. 1995; 98(4): 261–267. https://pubmed.ncbi.nlm.nih.gov/7636923/. 7636923

[18] Chandramohan D, Cutts F, Chandra R. Effects of a maternity waiting home on adverse maternal outcomes and the validity of antenatal risk screening. Int J Gynaecol Obstet. 1994; 46(3): 279–284. https://pubmed.ncbi.nlm.nih.gov/7805996/. DOI: 10.1016/0020-7292(94)90406-5 7805996

[19] Fogliati P, Straneo M, Mangi S, Azzimonti G, Kisika F, Putoto G. A new use for an old tool: Maternity waiting homes to improve equity in rural childbirth care. Results from a cross-sectional hospital and community survey in Tanzania. Health Policy Plan. December 1, 2017; 32: 1354–60. https://www.ncbi.nlm.nih.gov/pubmed/29040509. DOI: 10.1093/heapol/czx100 29040509 PMC5886146

[20] Meshesha B, Dejene G, Hailemariam T. The role of maternity waiting area in improving obstetric outcomes: A comparative cross-sectional study, Jinka Zonal Hospital, Southern Regional State. J Women’s Health Care. 2017; 6(6). https://www.researchgate.net/publication/322608729_The_Role_of_Maternity_Waiting_Area_in_Improving_Obstetric_Outcomes_A_Comparative_Cross-sectional_Study_Jinka_Zonal_Hospital_Southern_Regional_State.

[21] Lori JR, Boyd CJ, Munro-Kramer ML, et al. Characteristics of maternity waiting homes and the women who use them: Findings from a baseline cross-sectional household survey among SMGL-supported districts in Zambia. PLoS One. 2018; 13(12): e0209815. DOI: 10.1371/journal.pone.0209815 30596725 PMC6312364

[22] Lori JR, Perosky J, Munro-Kramer ML, et al. Maternity waiting homes as part of a comprehensive approach to maternal and newborn care: A cross-sectional survey. BMC Pregnancy Childbirth. 2019; 19: 228. DOI: 10.1186/s12884-019-2384-6 31272402 PMC6610940

[23] Tumwine JK, Dungare PS. Maternity waiting shelters and pregnancy outcome: experience from a rural area of Zimbabwe. Annals Trop Paediatrics. 1996; 6(1): 55–59. DOI: 10.1080/02724936.1996.11747804 8787367

[24] Singh K, Speizer I, Kim ET, Lemani C, Phoya A. Reaching vulnerable women through maternity waiting homes in Malawi. Int J Gynaecol Obstet. 2017; 136(1): 91–97. https://pubmed.ncbi.nlm.nih.gov/28099696/. DOI: 10.1002/ijgo.12013 28099696

[25] Singh, K, Speizer, IS, Kim, ET, Lemani C, Tang JH, Phoya A. Evaluation of a maternity waiting home and community education program in two districts of Malawi. BMC Pregnancy Childbirth. 2018; 18: 457. DOI: 10.1186/s12884-018-2084-7 30470256 PMC6251123

[26] Vermeiden T, Braat F, Medhin G, Gaym A, van den Akker T, Stekelenburg J. Factors associated with intended use of a maternity waiting home in Southern Ethiopia: A community-based cross-sectional study. BMC Pregnancy Childbirth. 2018; 18. DOI: 10.1186/s12884-018-1670-z 29351786 PMC5775531

[27] Chibuye PS, Bazant ES, Wallon M, Rao N, Fruhauf T. Experiences with and expectations of maternity waiting homes in Luapula Province, Zambia: A mixed-methods, cross-sectional study with women, community groups and stakeholders. BMC Pregnancy Childbirth. 2018; 18(1): 42. DOI: 10.1186/s12884-017-1649-1 29370773 PMC5785796

[28] McIntosh N, Gruits P, Oppel E, Shao A. Built spaces and features associated with user satisfaction in maternity waiting homes in Malawi. Midwifery. 2018; 62: 96–103. DOI: 10.1016/j.midw.2018.03.020 29660576

[29] Suwedi-Kapesa LC, Nyondo-Mipando AL. Assessment of the quality of care in Maternity Waiting Homes (MWHs) in Mulanje District, Malawi. Malawi Med J. 2018; 30(2): 103–110. https://www.ncbi.nlm.nih.gov/pmc/articles/PMC6307072/. DOI: 10.4314/mmj.v30i2.10 30627338 PMC6307072

[30] Garcia Prado A, Cortez R. Maternity waiting homes and institutional birth in Nicaragua: policy options and strategic implications. Int J Health Plann Manage. 2012; 27(2): 150–166. https://onlinelibrary.wiley.com/doi/abs/10.1002/hpm.1107. DOI: 10.1002/hpm.1107 22052420

[31] Kruk ME, Mbaruku G, Rockers PC, Galea S. User fee exemptions are not enough: out-of-pocket payments for “free” delivery services in rural Tanzania. Trop Med Int Health. 2008; 13(12): 1442–1451. DOI: 10.1111/j.1365-3156.2008.02173.x 18983268

[32] Kruk ME, Mbaruku G, McCord CW, Moran M, Rockers PC, Galea S. Bypassing primary care facilities for childbirth: A population-based study in rural Tanzania. Health Pol Plann. 2009; 24(4): 279–288. https://academic.oup.com/heapol/article/24/4/279/567082. DOI: 10.1093/heapol/czp011 19304785

[33] Ngoma-Hazemba A, Hamomba L, Silumbwe A, Munakampe MN, Soud F. Community Perspectives of a 3-Delays Model Intervention: A Qualitative Evaluation of Saving Mothers, Giving Life in Zambia. Glob Health Sci Pract. 2019; 7(Suppl 1): S139–S150. https://www.ncbi.nlm.nih.gov/pmc/articles/PMC6519671/. DOI: 10.9745/GHSP-D-18-00287 30867214 PMC6519671

[34] Ngoma T, Asiimwe AR, Mukasa J, et al. Addressing the Second Delay in Saving Mothers, Giving Life Districts in Uganda and Zambia: Reaching Appropriate Maternal Care in a Timely Manner. Glob Health Sci Pract. 2019; 7(Suppl 1): S68–S84. https://www.ncbi.nlm.nih.gov/pmc/articles/PMC6519669/. DOI: 10.9745/GHSP-D-18-00367 30867210 PMC6519669

[35] van den Heuvel OA, de Mey WG, Buddingh H, Bots ML. Use of maternal care in a rural area of Zimbabwe: A population-based study. Acta Obstet Gynecol Scand. 1999; 78(10): 838–46. DOI: 10.1034/j.1600-0412.1999.781002.x 10577611

[36] Scott NA, Vian T, Kaiser JL, et al. Listening to the community: Using formative research to strengthen maternity waiting homes in Zambia. PLoS One. 2018; 13. DOI: 10.1371/journal.pone.0194535 PMC5854412 29543884

[37] Selbana DW, Derese M, Sewmehone Endalew E, Gashaw BT. A Culturally Sensitive and Supportive Maternity Care Service Increases the Uptake of Maternity Waiting Homes in Ethiopia. Int J Womens Health. 2020; 12: 813–821. https://www.ncbi.nlm.nih.gov/pmc/articles/PMC7553138/. DOI: 10.2147/IJWH.S268245 33116931 PMC7553138

[38] Endayehu M, Yitayal M, Debie A. Intentions to use maternity waiting homes and associated factors in Northwest Ethiopia. BMC Pregnancy Childbirth. 2020; 20(1): 281. https://www.ncbi.nlm.nih.gov/pmc/articles/PMC7216713/. DOI: 10.1186/s12884-020-02982-0 32393188 PMC7216713

[39] Gezimu W, Bitewa YB, Tesema MT, Wonde TE. Intention to use maternity waiting home and associated factors among pregnant women in Gamo Gofa zone, Southern Ethiopia, 2019. PLoS One. 2021; 16(5): e0251196. https://pubmed.ncbi.nlm.nih.gov/33983992/. DOI: 10.1371/journal.pone.0251196 33983992 PMC8118329

[40] Saati H, McLaughlin MM, Seung KJ. The role of maternity waiting homes as part of a comprehensive maternal mortality reduction strategy in Lesotho. PIH Reports [Internet]. 2013; 1(1). https://www.pih.org/sites/default/files/2017-07/PIH_Report_Sept_IndividualPgs.pdf.

[41] Lori JR, Perosky JE, Rominski S, et al. Maternity waiting homes in Liberia: Results of a countrywide multi-sector scale-up. PLoS One. 2020; 15(6). DOI: 10.1371/journal.pone.0234785 PMC7310707 32574182

[42] Henry EG, Ngoma T, Kaiser JL, et al. Evaluating implementation effectiveness and sustainability of a maternity waiting homes intervention to improve access to safe delivery in rural Zambia: A mixed-methods protocol. BMC Health Serv Res. 2020; 20. DOI: 10.1186/s12913-020-4989-x 32164728 PMC7068884

[43] Andemichael G, Haile B, Kosia A, Mufunda J. Maternity waiting homes: A panacea for maternal/neonatal conundrums in Eritrea. JEMA. 2009; 4(1). https://www.ajol.info//index.php/jema/article/view/52112. DOI: 10.4314/jema.v4i1.52112

[44] Vermeiden T, Schiffer R, Langhorst J, et al. Facilitators for maternity waiting home utilization at Attat Hospital: A mixed-methods study based on 45 years of experience. Trop Med Int Health. 2018; 23(12): 1332–1341. DOI: 10.1111/tmi.13158 30286267

[45] Perosky JE, Munro-Kramer ML, Lockhart N, Musonda GK, Naggayi A, Lori JR. Maternity waiting homes as an intervention to increase facility delivery in rural Zambia. Int J Gynaecol Obstet. 2019; 146(2): 266–267. DOI: 10.1002/ijgo.12864 31099092

[46] James KH, Perosky JE, McLean K, Nyanplu A, Moyer CA, Lori JR. Protocol for geolocating rural villages of women in Liberia utilizing a maternity waiting home. BMC Res Notes. 2019; 12(1): 196. https://www.ncbi.nlm.nih.gov/pmc/articles/PMC6444816/. DOI: 10.1186/s13104-019-4224-1 30940187 PMC6444816

[47] Buser JM, Munro-Kramer ML, Carney M, Kofa A, Cole GG, Lori JR. Maternity waiting homes as a cost-effective intervention in rural Liberia. Int J Gynaecol Obstet. 2019; 146(1): 74–79. https://pubmed.ncbi.nlm.nih.gov/31026343/. DOI: 10.1002/ijgo.12830 31026343

[48] Buser JM, Munro-Kramer ML, Veliz PT, et al. How maternity waiting home use influences attendance of antenatal and postnatal care. PLoS One. 2021; 16(1): e0245893. https://www.ncbi.nlm.nih.gov/pmc/articles/PMC7822518/. DOI: 10.1371/journal.pone.0245893 33481942 PMC7822518

[49] Scott NA, Vian T, Kaiser JL, et al. Listening to the community: Using formative research to strengthen maternity waiting homes in Zambia. PLoS One. 2018; 13(3): e0194535. https://www.ncbi.nlm.nih.gov/pmc/articles/PMC5854412/. DOI: 10.1371/journal.pone.0194535 29543884 PMC5854412

[50] Bonawitz R, McGlasson KL, Kaiser JL, et al. Quality and utilization patterns of maternity waiting homes at referral facilities in rural Zambia: A mixed-methods multiple case analysis of intervention and standard of care sites. PLoS One. 2019; 14(11): e0225523. https://www.ncbi.nlm.nih.gov/pmc/articles/PMC6881034/. DOI: 10.1371/journal.pone.0225523 31774838 PMC6881034

[51] Kebede KM, Mihrete KM. Factors influencing women’s access to the maternity waiting home in rural Southwest Ethiopia: A qualitative exploration. BMC Pregnancy Childbirth. 2020; 20. DOI: 10.1186/s12884-020-02988-8 32408875 PMC7226938

[52] Lori JR, Wadsworth AC, Munro ML, Rominski S. Promoting access: The use of maternity waiting homes to achieve safe motherhood. Midwifery. 2013; 29(10): 1095–1102. https://www.ncbi.nlm.nih.gov/pmc/articles/PMC3787070/. DOI: 10.1016/j.midw.2013.07.020 24012018 PMC3787070

[53] Lori JR, Munro-Kramer ML, Mdluli EA, Musonda GK, Boyd CJ. Developing a community driven sustainable model of maternity waiting homes for rural Zambia. Midwifery. 2016; 41: 89–95. https://www.sciencedirect.com/science/article/pii/S0266613816301395. DOI: 10.1016/j.midw.2016.08.005 27571773

[54] Fontanet CP, Fong RM, Kaiser JL, et al. A qualitative exploration of community ownership of a maternity waiting home model in rural Zambia. Global Health Sci Pract. 2020; 8(3). www.ghspjournal.org. DOI: 10.9745/GHSP-D-20-00136 PMC7541113 33008852

[55] Nigussie T, Yaekob R, Geremew M, Asefe A. Predictors of intention to use maternity waiting home among pregnant women in Bench Maji Zone, Southwest Ethiopia using the Theory of Planned Behavior. Int J Women Health. 2020; 12. DOI: 10.2147/IJWH.S267730 PMC7602906 33149701

[56] Spaans WA, van Roosmalen J, van Wiechen CM. A maternity waiting home experience in Zimbabwe. Int J Gynaecol Obstet. 1998; 61(2): 179–80. https://pubmed.ncbi.nlm.nih.gov/9639223/. DOI: 10.1016/S0020-7292(98)00027-7 9639223

[57] Getachew B, Liabsuetrakul T. Health care expenditure for delivery care between maternity waiting home users and nonusers in Ethiopia. Int J Health Plann Manage. 2019; 34(2): e1334–e1345. https://pubmed.ncbi.nlm.nih.gov/30924204/. DOI: 10.1002/hpm.2782 30924204

[58] Bergen N, Abebe L, Asfaw S, et al. Maternity waiting areas – serving all women? Barriers and enablers of an equity-oriented maternal health intervention in Jimma Zone, Ethiopia. Glob Public Health. 2019; 14(10): 1509–1523. DOI: 10.1080/17441692.2019.1597142 30905270

[59] Ruiz MJ, van Dijk MG, Berdichevsky K, Munguía A, Burks C, García SG. Barriers to the use of maternity waiting homes in indigenous regions of Guatemala: A study of users’ and community members’ perceptions. Cult Health Sex. 2013; 15(2): 205–18. DOI: 10.1080/13691058.2012.751128 23234509

[60] Martey JO, Djan JO, Twum S, Browne EN, Opuku SA. Utilisation of maternal health services in Ejisu District, Ghana. West Afr J Med. 1995; 14(1): 24–28. https://europepmc.org/article/med/7626528. 7626528

[61] Wilson JB, Collison AHK, Richardson D, Kwofie G, Senah KA, EK Tinkorang. The maternity waiting home concept: the Nsawam, Ghana experience. Int J Gynaecol Obstet. 1998; 59: S165–172. DOI: 10.1016/S0020-7292(97)00162-8 9389628

[62] Mramba L, Nassir FA, Ondieki C, Kimanga D. Reasons for low utilization of a maternity waiting home in rural Kenya. Int J Gynaecol Obstet. 2010; 108: 152–160. https://www.researchgate.net/publication/38069854_Reasons_for_low_utilization_of_a_maternity_waiting_home_in_rural_Kenya. DOI: 10.1016/j.ijgo.2009.08.029 19892347

[63] Eckermann E, Deodato G. Maternity waiting homes in Southern Lao PDR: The unique “silk home.” J Obstet Gynaecol Res. 2008; 34(5): 767–75. https://pubmed.ncbi.nlm.nih.gov/18834334/. DOI: 10.1111/j.1447-0756.2008.00924.x 18834334

[64] Buser JM, Moyer CA, Boyd CJ, et al. Maternal knowledge of essential newborn care in rural Zambia. Health Care Women Int. 2020; 13: 1–16. https://pubmed.ncbi.nlm.nih.gov/32658563/. 10.1080/07399332.2020.1781125 32658563

[65] Wild K, Barclay L, Kelly P, Martins N. The tyranny of distance: Maternity waiting homes and access to birthing facilities in rural Timor-Leste. Bull World Health Organ. 2012; 90(2): 97–103. https://www.ncbi.nlm.nih.gov/pmc/articles/PMC3302550/. DOI: 10.2471/BLT.11.088955 22423160 PMC3302550

[66] Wild K, Kelly P, Barclay L, Martins N. Agenda setting and evidence in maternal health: connecting research and policy in Timor-Leste. Front Public Health. 2015; 10. https://www.frontiersin.org/articles/10.3389/fpubh.2015.00212/full. DOI: 10.3389/fpubh.2015.00212 PMC4564655 26442239

[67] Sialubanje C, Massar K, van der Pijl MS, Kirch EM, Hamer DH, Ruiter RA. Improving access to skilled facility-based delivery services: Women’s beliefs on facilitators and barriers to the utilisation of maternity waiting homes in rural Zambia. Reprod Health. 2015; 12: 61. https://www.ncbi.nlm.nih.gov/pmc/articles/PMC4493824/. DOI: 10.1186/s12978-015-0051-6 26148481 PMC4493824

[68] Sialubanje C, Massar K, Hamer DH, et al. Personal and environmental factors associated with the utilisation of maternity waiting homes in rural Zambia. BMC Pregnancy Childbirth. 2017; 17: 136. https://bmcpregnancychildbirth.biomedcentral.com/articles/10.1186/s12884-017-1317-5#citeas. DOI: 10.1186/s12884-017-1317-5 28472945 PMC5418767

[69] Jacobson JL. Maternal mortality and morbidity. Zimbabwe’s birth force. Newsl Womens Glob Netw Reprod Rights. 1991; 36: 16–17. https://pubmed.ncbi.nlm.nih.gov/12284525/. 12284525

[70] Jacobson JL. Zimbabwe’s birth force. World Watch. 1991; 4(4): 5–6. https://pubmed.ncbi.nlm.nih.gov/12343752/. 12343752

[71] Vian T, White EE, Biemba G, Mataka K, Scott N. Willingness to Pay for a Maternity Waiting Home Stay in Zambia. J Midwifery Womens Health. 2017; 62(2): 155–162. https://www.ncbi.nlm.nih.gov/pmc/articles/PMC5836912/. DOI: 10.1111/jmwh.12528 28419708 PMC5836912

[72] Kaiser JL, Fong RM, Ngoma T, et al. The effects of maternity waiting homes on the health workforce and maternal health service delivery in rural Zambia: A qualitative analysis. Hum Resour Health. 2019; 17: 93. DOI: 10.1186/s12960-019-0436-7 31801578 PMC6894259

[73] Tiruneh GT, Getu YN, Abdukie MA, Eba GG, Keyes E, Bailey PE. Distribution of maternity waiting homes and their correlation with perinatal mortality and direct obstetric complication rates in Ethiopia. BMC Pregnancy Childbirth. 2019; 19(1): 214. https://www.ncbi.nlm.nih.gov/pmc/articles/PMC6593553/. DOI: 10.1186/s12884-019-2356-x 31238909 PMC6593553

[74] Figa-Talamanca. Maternal mortality and the problem of accessibility to obstetric care; The strategy of maternity waiting homes. Soc Sci Med. 1996; 42(10): 1381–1390. https://www.sciencedirect.com/science/article/abs/pii/0277953695002863?via%3Dihub. DOI: 10.1016/0277-9536(95)00286-3 8735894

[75] Bøhler E. Maternity waiting homes – an effective instrument for global maternal health. Tidsskr Nor Laegeforen. 2018; 138(5). https://pubmed.ncbi.nlm.nih.gov/29513469/. DOI: 10.4045/tidsskr.17.0717 29513469

[76] Danilack VA, Nunes AP, Phipps MG. Unexpected complications of low-risk pregnancies in the United States. Am J Obstet Gynecol. 2015; 212(6): 809.e1–809.e8096. https://www.ncbi.nlm.nih.gov/pmc/articles/PMC4728153/. DOI: 10.1016/j.ajog.2015.03.038 26042957 PMC4728153

[77] Kurji J. Gebretsadik LA, Wordofa MA, et al. Effectiveness of upgraded maternity waiting homes and local leader training on improving institutional births: A cluster-randomized controlled trial in Jimma, Ethiopia. BMC Public Health. 2020; 20. DOI: 10.1186/s12889-020-09692-4 33092565 PMC7583173

