# Supplementary material for: Maternity Waiting Home Interventions as a Strategy for Improving Birth Outcomes: A Scoping Review and Meta-Analysis
Source: Ann Glob Health. 2022 Jan 18;88(1):8. doi: 10.5334/aogh.3496 (PMC8782095; doi:10.5334/aogh.3496)
Supplement: Appendix 1. — TMWH Literature Review Summary Table. [file agh-88-1-3496-s1.pdf]

Appendix 1: MWH Literature Review Summary Table

First author, title (year)	Location	Research objective	Study design	Sample Size	Key findings	Implications/ Recommendations
Poovan, A maternity waiting home reduces obstetric catastrophes (1990)	Ethiopia (Africa)	Assess the impact of Attat Hospital MWH *(tukul)* on maternal and perinatal mortality.	Retrospective cohort study comparing women who used *tukul* and direct hospital admissions	N = 142 *tukul* deliveries N = 635 direct admit deliveries	- A higher proportion of women in the *tukul* group delivered by C-section (72%) than those directly admitted to the hospital (25%).<br>- No ruptured uteri nor craniotomies among women who utilized the *tukul*, while 45 in the direct admission group had a ruptured uterus and 23 delivered by craniotomy<br>- 13 maternal deaths among direct admissions, no maternal deaths among *tukul* users<br>- Stillbirth rate among direct admissions (253.5 per 1,000 births) was 10x higher than that of the *tukul* group (21.2 per 1,000 births)	- MWHs close to a rural hospital are crucial for improving maternal and perinatal health outcomes for women with high-risk pregnancies, and women who live a long distance away from the hospital<br>- An effective maternal service needs informed community participation, special attention to antenatal care, a referral system, and an MWH
Millard, Antenatal village stay and pregnancy outcome in rural Zimbabwe (1991)	Zimbabwe (Africa)	Compare the pregnancy outcomes of those who stayed at the antenatal village (ANV) in Mt. Selinda and those who were admitted directly to the Willis Pierce Hospital.	Retrospective cohort study comparing antenatal village (ANV) users and direct hospital admissions	N = 502 births by women who stayed at the ANV N = 352 births by direct admissions	- The ANV group had more women with an absence of antenatal risk factors (83.7%) than the direct admission group (77.7%)<br>- Perinatal mortality was lower in the ANV group (35 per 1,000 births) than the direct admit group (71 per 1,000 births)<br>- Fewer assisted deliveries in the ANV group (14.2%) than the direct admit group (23.2%)<br>- No significant difference for	- Better perinatal outcomes and lower rates of obstetrical intervention were found in women staying at the ANV<br>- It is possible that differences in antenatal risk factors, or unmeasured differences such as SES, explained some or all of the differences in pregnancy outcomes<br>- Recommends

					duration of labor, length of hospital admission, or post-partum hemorrhage	intention-to-treat methodology to provide a more definitive answer to the efficacy of ANVs
van Lonkhuijzen, Use of maternity waiting home in rural Zambia (2003)	Zambia (Africa)	Assess the results from the use of a MWH at Nyanjie RCZ Hospital in rural Zambia between May and Nov 1994.	Prospective cohort study comparing MWH users and non-users who gave birth at a hospital in rural Zambia	N = 218 MWH users<br>N = 292 non-MWH users	- Women in the MWH group were significantly more likely to have a history of a prior C-section (7% vs 2%, p<0.01)<br>- MWH users had significantly higher rates of breech and transverse presentations (12% vs 7%, p<0.05)<br>- Significantly more women in the MWH group suffered from hypertension during birth (6% vs 1%, p< 0.001)<br>- No difference in perinatal or maternal mortality between the two groups, although the MWH group had more high-risk pregnancies<br>- No difference in mean birth weight or percentage of low birth weight children between the two groups	- MWH success dependent on properly functioning referral system<br>- Difficult to draw conclusions on MWH effectiveness by comparing two groups delivering in the same hospital – room for unknown bias<br>- A better study would compare pregnancy outcomes in two separate communities, one with a MWH and one without – ideally assign women at random to a MWH in a large community
Andemichael, Maternity waiting homes: A panacea for maternal/ neonatal conundrums in Eritrea (2009)	Eritrea (Africa)	Assess pregnancy outcomes (maternal and perinatal mortality) before and after establishment of MWHs in remote regions of Eritrea.	Pre- and post-intervention study, data collected 1-year pre-MWH and 2 years post-MWH	N = 266 pre-MWH deliveries,<br>N = 862 post-MWH deliveries	- 5 maternal deaths pre-MWH (1.9%)<br>- no maternal deaths post-MWH<br>- Perinatal mortality rate remains high at 1.6%, but is better than other developing countries<br>- All stillbirths and neonatal deaths after the implementation of MWHs occurred in mothers who came to the hospital after many hours of labor and failed attempt to deliver at home	- MWHs contribute to the increased in skilled attendance during pregnancy and childbirth, and thus pregnancy outcomes in this region<br>- MWHs should be staffed by qualified health workers with continuous refresher courses and on-the-job training<br>- TBAs and local

						administration play key role in bringing mothers to MWHs
Kelly, The role of a maternity waiting area (MWA) n reducing maternal mortality and stillbirths in high-risk women in rural Ethiopia (2010)	Ethiopia (Africa)	Investigate the frequency of maternal mortality and stillbirths among women who were admitted to Attat Hospital via the attached MWA and those who were directly admitted to the same hospital.	Retrospective cohort study comparing women who used the MWA to direct hospital admissions	N = 6805 MWA users<br>N = 17,343 non-MWA users	- MMR was 89.9 per 100,000 live births for MWA users, and 1333.1 per 100,000 live births for non-MWA users<br>- Higher proportion of MWA users had a C-section (38.5%) than non-users (20.3%)<br>- No complete uterine ruptures occurred in the MWA group, compared to 5.8% of non-MWA users (cause of 33.2% of deaths in non-MWA group)<br>- Stillbirth rate for MWA users was 17.6 per 1,000 births, compared with 191.2 per 1,000 births in non-MWA users<br>- There were a higher proportion of young mothers (45% vs 37%) and first-time mothers (48% vs 37%) in the non-MWA group than the MWA group	- MWA users were more likely to be high-risk, as shown by the higher rates of delivery by C-section<br>- Cost of transport was a major cause of delay in seeking care, and thus contributed to maternal mortality and stillbirth among non-MWH users<br>- Attribute success of Attat MWH to its strong community links and acceptability to women and their husbands, community opinion is sought for any new building or project commences<br>- While a randomized controlled trial would have been better for assessing the effectiveness of MWAs, it was not ethically possible in this community because it is already accepted as being beneficial, but could be implemented in other locations
Gaym, Maternity waiting homes in Ethiopia – three decades	Ethiopia (Africa)	Describe the infrastructural settings, service utilization, admission indications,	Retrospective cohort study comparing women who used the MWH to	N = 902 MWH users<br>N = 3373 non-MWH users	- St. Luke's Hospital MWH: 6 stillbirths among MWH users (2.3%) vs 165 among non-users (7.3%), 33% of MWH mothers had C-	- Mothers who utilized MWHs had fewer stillbirths, maternal deaths, instrumental deliveries, and

experience (2012)		distances travelled, duration of stay, maternal and perinatal outcomes, and mothers' opinions of the MWHs.	direct hospital admissions at two hospitals (St. Luke's and Attat)		sections compared to 20%, no MWH maternal deaths vs 9 non-MWH maternal deaths<br>- Attat Hospital MWH: stillbirth rate was 1.2% among MWH users vs 10%, instrumental delivery rate was 19.3% among MWH users vs 29%, 40.6% of MWH mothers had C-sections vs 20%, no MWH maternal deaths vs 3 non-MWH maternal deaths<br>- Challenges: economic barriers (clients are responsible for their own food and other supplies), social/family obligations at home, disparities due to different levels of support mothers received, lack of facilities for families to stay	craniotomies than those who did not use a MWH<br>- MWH mothers had higher rates of C-sections than non-MWH mothers<br>- There are social, economic, and geographic barriers that prevent women who need MWH services from using them<br>- Future MWHs should be established at more rural health clinics with ambulance services to hospitals so that mothers in more remote regions can access them<br>- MWHs need to formally standardize and institutionalize admission, care, and discharge protocols, staff assignments, and service statistics/ data collection
Lori, Maternity waiting homes and traditional midwives in rural Liberia (2013)	Liberia (Africa)	Determine the effectiveness of MWHs in increasing the use of SBAs and decreasing maternal and child morbidity and mortality and to understand the changing role of TMs.	Pre- and post-intervention analysis conducted halfway through a large prospective cohort study comparing 10 rural Liberian communities, 5 of which received the intervention (establishment of MWH)	N = 8,477 MWH users<br>N = 9,567 non-MWH users	- Communities where a MWH was established had lower rates of maternal and perinatal death and higher rates of team births than those without one<br>- 3 maternal deaths among MWH communities (0.035%) vs 12 among non-MWH communities (0.125%)<br>- 43 perinatal deaths among MWH communities (0.51%) vs 60 in non-MWH	- The MWHs in this study had positive outcomes in maternal and perinatal mortality as well as proportion of team births, and should be included in Liberia's plan to reduce maternal and neonatal morbidity and mortality<br>- TMs are an integral part of the birthing team, as they can address many of the

			and 5 did not (control)		communities (0.63%)<br>- 95.2% team births in MWH communities vs 69.8% in non-MWH communities (both communities saw an increase in team births over the 2-year study)<br>- TMs considered the MWHs to be helpful to mothers and to themselves, and were happy to bring women to the MWH to rest before giving birth<br>- TMs felt the MWH environment and increased collaboration between themselves and certified midwives facilitated safer deliveries in contrast to the traditional at-home birth experience of rural Liberian women	barriers to women in rural low-income settings accessing MWHs (lack of knowledge, location, cost, and cultural barriers)
Braat, Comparison of pregnancy outcomes between maternity waiting home users and non-users at hospitals with and without a maternity waiting home: retrospective cohort study (2018)	Ethiopia (Africa)	Examine the impact of a MWH by comparing pregnancy outcomes between women who did and did not utilize the MWH at Attat Hospital, which has a MWH, and Butajura Hospital, which does not have one.	Retrospective cohort study comparing the pregnancy outcomes of MWH users to non-MWH users at the same hospital, and women who gave birth in a hospital without an MWH	N = 2,784 births among MWH users<br>N = 14,895 births among non-MWH users	- MWH users were less educated, poorer, and had further to travel compared with non-users<br>- No maternal deaths occurred in the MWH group at Attat, compared with 20 (0.4%) in the non-MWH group at Attat, and 31 (0.3%) at Butajira<br>- 38 stillbirths (1.4%) among MWH users, 393 (7.2%) among non-users at Attat, and 717 (7.2%) at Butajira<br>- MWH users had highest proportion of C-sections (41.1%), compared with 22.0% among non-users at Attat and	- Despite facing more obstacles and having higher risk pregnancies, the more vulnerable group of women who utilized the MWH had better birth outcomes than the higher SES women who did not utilize a MWH<br>- All maternal deaths and nearly all stillbirths and uterine ruptures occurred among non-MWH users<br>- Higher C-section rates in MWH group indicative of high-risk status of pregnancies<br>- MWH reaching

					17.9% at Butajira<br>- MWH users also had highest proportion of assisted vaginal deliveries (20.9%), compared with 13.5% among non-users at Attat and 11.7% at Butajira	rural, poor, uneducated, high-risk women may be the result of an extensive community health promotion plan<br>- RCT would be ethically challenging, recommend comparing outcomes of home and facility boths in communities with and without a MWH
Fogliati, A new use for an old tool maternity waiting homes to improve equity in rural childbirth care (2017)	Tanzania (Africa)	Determine whether MWHs may be a strategy to improve access to facilities for delivery for poor women.	Secondary analysis of cross-sectional survey comparing women who utilized an MWH and those who were admitted to the hospital directly in Tanzania	N = 348 births among MWH users<br>N = 729 births among non-MWH users	- The women who utilized the MWH tended to be less educated, poorer, and live further away from the birthing facility than those who did not<br>- 12 (3.4%) stillbirths in the MWH group, 21 (2.9%) in the non-MWH group<br>- 3 (0.9%) neonatal deaths in the MWH group, 22 (3.1%) in the non-MWH group<br>- 14 (4.0%) perinatal deaths in the MWH group, 41 (5.6%) perinatal deaths in the non-MWH group<br>- A total of 6 maternal deaths were recorded, with one in the MWH group and 4 in the non-MWH group (MWH info was not available for one of the deaths)	- MWHs are an important tool for allowing women who cannot afford emergency transportation to give birth in a hospital, improving equity in childbirth related outcomes<br>- Distance from the hospital is a major factor in MWH utilization, suggesting it is a good intervention for rural populations
Meshesha, The role of maternity waiting area in improving obstetric outcomes: a comparative cross-sectional	Ethiopia (Africa)	Assess the role of the MWA at Jinka Zonal Hospital in improving pregnancy outcomes for the reference of the	Hospital-based comparative cross-sectional study of women who gave birth at Jinka Zonal Hospital who	N = 86 MWA-admitted mothers<br>N = 430 direct hospital admissions	- The mean number of bad obstetric outcomes was significantly lower in the MWA-admitted mothers compared to those admitted from home<br>- Mothers who came directly from home	- MWA-admission had a strong protective association with all of the bad obstetric outcomes: MWA mothers had significantly lower prevalence of bad

study, Jinka Zonal Hospital, Southern Regional State (2017)		hospital and other MWAs in the region.	did and did not utilize the MWA		experienced 3-7 bad obstetric outcomes at the same time whereas the MWA mothers had a maximum of two bad obstetric outcomes at the same time on the same mother<br>- MWA-admitted mothers were 97% less likely to have prolonged PROM than mothers from home<br>- MWA mothers were 75% less likely to experience delay in admission to the labor ward when in labor compared to those who came in from home<br>- MWA increased likelihood of operative delivery (statistically insignificant when adjusted for confounders)	outcomes (33.7%) than mothers who came from home (61.2%) –overall improvement in obstetric outcomes by 27.5% with admission to the MWA<br>- The majority of the mothers who utilized the MWA were from rural areas<br>- Pregnant mothers from rural areas had statistically significant higher odds of experiencing bad obstetric outcomes compared to urban mothers
Lori, Maternity waiting homes in Liberia: Results of a countrywide multi-sector scale-up (2020)	Liberia (Africa)	To describe the evolutionary development of MWHs as a component of the larger health system strengthening efforts; describe the role of MWHs to improve maternal health from a community and healthcare provider viewpoint; and document the successes,	A convergent parallel mixed methods study examining multiple aspects of MWH scale-up across Liberia	N = 1179 focus-group participants (community members, women of reproductive age, traditional birth attendants, current MWH residents); N = 119 facilities examined	- In the years since the original construction of five MWHs, an additional 114 MWHs were constructed in 14 of the 15 counties in Liberia.<br>- Monthly stays at facilities funded by community were 2.5 times those funded by NGOs (IRR, 2.46, 95% CI 1.33–4.54).<br>- Attributes of	- Success factors for scale-up and sustainability included strong government support through development of public policy, local and county leadership, early and sustained engagement with communities, and

		challenges, and barriers to sustainability and scale-up of MWHs.			sustainability included strong local leadership/active community engagement and community ownership and governance.<br>- Community members and healthcare providers generally believed that MWHs are effective in reducing home delivery, reducing maternal deaths, and improving relationships between facility staff, TBAs, and communities.	self-governance.<br>- A multi-pronged approach with strong community engagement is key to the scale-up and sustainability of MWHs as an intervention to increase facility delivery for women living the farthest from a healthcare facility.<br>- MWH scale-up should continue as a component of comprehensive health system strengthening.
Henry, Evaluating implementation effectiveness and sustainability of a maternity waiting homes intervention	Zambia (Africa)	The overall aim of this study is to generate evidence on the implementation effectiveness and sustainability of the	A mixed-methods approach involving longitudinal, cross-sectional data collection, guided by the Consolidated	N = 10 rural intervention sites; N = 10 control sites	Data collection for this project is ongoing.	- The findings from this evaluation will be shared with policymakers formulating policy affecting

to improve access to safe delivery in rural Zambia: A mixed-methods protocol (2020)		Maternity Homes Access in Zambia (MAHMAZ) project and reasons for variation in order to inform the interpretation of the outcomes of the main trial.	Framework for Implementation Research (CFIR)				the implementation of MWH and may be used as evidence for programmatic decisions by the government and supporting agencies in deciding to take this model to scale.
Kurji, Effectiveness of upgraded maternity waiting homes and local leader training on improving institutional births: a cluster-randomized controlled trial in Jimma, Ethiopia (2020)	Ethiopia (Africa)	To evaluate the effectiveness of upgraded MWHs and local leader training in improving institutional births in Jimma Zone, Ethiopia	A pragmatic, three-arm, stratified, cluster-randomized trial design was used to evaluate the effect of upgraded, functional MWHs (MWH+) and leader training on the primary outcome of institutional births.	24 clusters of 160 women each per round of surveys; N = 7593 total respondents	- Data from 24 PHCUs and 7593 women were analysed using intention-to-treat. The proportion of institutional births was comparable at baseline between the three arms.<br>- At endline, institutional births were slightly higher in the MWH+ training (54% [n = 671/1239]) and training only arms (65% [n = 821/1263]) compared to usual care		- The combination of upgraded MWHs and leader training lead to small but non-significant improvements in institutional birth levels in Jimma Zone.<br>- Low MWH use has been linked to the poor quality of services offered. Only 15% of women

						(51% [n = 646/ 1271]). MWH use at baseline was 6.7% (n = 256/3784) and 5.8% at endline (n = 219/3809).<br>- Both intervention groups exhibited a non-statistically significant higher odds of institutional births compared to usual care (MWH+ &leader training odds ratio [OR] = 1.09, 97.5% confidence interval [CI] 0.67 to 1.75; leader training OR = 1.37, 97.5% CI 0.85 to 2.22)	in endline from the MWH+ training arm who did not use MWHs said it was because they were dissatisfied with the quality of services.<br>- Another important reason for low MWH use among some women may have been a relative short distance between homes and health facilities, making direct access to the facility possible.
Kebede, Factors influencing women's access to the maternity waiting home in rural Southwest Ethiopia: a qualitative	Ethiopia (Africa)	To explore the factors influencing women's access to MWHs in Southwest Ethiopia by applying the 'A-framework' of access	A qualitative approach using focus groups and in-depth interviews was employed. The rationale for this approach is to explore	A total of 4 FGDs and 18 IDIs (5 with clinicians, 6 with HEWs and 7 with MWH non-users) were		- Women had interest on MWHs and are aware of the existence of MWHs in their immediate vicinity. Health information	- The factors influencing women's access to the MWHs were structural and individua

exploration (2020)		proposed by Thiede et al.	more in-depth about factors influencing women’s access to the MWHs.	conducted.	disseminations and referral linkages by frontline health workers enabled women to timely access the MWHs.<br>- Many women didn’t understand the aims and benefits of MWHs. At the facility level, there were attempts to improve the acceptability of MWHs by allowing women to choose their delivery positions. But, participants claimed lack of privacy and presence of disrespectful care.<br>- Physical barriers were considered as potential problems for women residing in remote areas. MWH users also mentioned absences of sufficient basic facilities,	l and resonate with Thiede et al.’s dimensions of access.<br>- A better understanding of which factors are most influential in preventing women’s access to the MWHs in rural Southwest Ethiopia is needed to appropriately target interventions.

						poor quality and varieties of food.	
Fontanet, A Qualitative Exploration of Community Ownership of a Maternity Waiting Home Model in Rural Zambia (2020)	Zambia (Africa)	To qualitatively explore how different stakeholders perceived community ownership of the MWH and how this changed over the first 24 months of MWH operations.	Investigators carried out 42 focus group discussions with community stakeholders and 161 in-depth interviews with MWH stakeholders at multiple time-points over 24 months. A content analysis and triangulation of findings were also completed to understand community ownership of the MWH and observe changes in perceptions of ownership over time.	N = 412 respondents across FGDs and IDIs.		- Community members' perceptions of ownership were related to their ability to use the MWH and a responsibility toward its success. Community and MWH stakeholders described increasingly more specific responsibilities over time.<br>- Governance committee and management unit members perceived their ability to represent the community as a crucial component of their role. Multiple respondent types saw collaboration between the governance committee and the health facility staff as key to allowing the MWH to meet its goal of	- The perceptions of community ownership evolved as the intervention became more established over time.<br>- Use of the MWH, and clear understanding of roles and responsibilities in management of the MWH, seemed to foster feelings of community ownership.<br>- To improve the sustainability of community-based maternal and child health programs, interventions should be accessible to target

						serving the community.	communities and clear roles should be established among stakeholders.
Nigussie, Predictors of Intention to Use Maternity Waiting Home Among Pregnant Women in Bench Maji Zone, Southwest Ethiopia Using the Theory of Planned Behavior (2020)	Ethiopia (Africa)	To investigate the predictors of behavioral intention toward MWH using the theory of planned behavior so as to fill the dearth of evidence on this important issue.	A community-based cross-sectional study was conducted from March 15 to June 20, 2018 in Bench Maji Zone, Southwest Ethiopia. A multistage sampling technique was used. The data were collected by trained data collectors using a structured pre-tested questionnaire.	N = 829 respondents in catchment area	-	A total of 829 women were interviewed. The mean age of respondents was 27.1 (±5.2) years. Thirty-nine percent of the respondents used maternity waiting home previously. The attitude (β =0.12, p<0.001), subjective norm (β =0.47, p<0.001), perceived behavioral control (β =0.42, p<0.001), and ANC use during current pregnancy (β =0.07, p=0.030) were predictors of intension to use maternity waiting homes. R square was calculated to be 81%.	The intention to use maternity waiting homes was significantly associated with antenatal care use, attitude, subjective norm, and perceived behavioral control. Thus, multi-dimensional interventions are important to increase the intension to use MWH.
