# Supplementary material for: Maternity Waiting Home Interventions as a Strategy for Improving Birth Outcomes: A Scoping Review and Meta-Analysis
Source: Ann Glob Health. 2022 Jan 18;88(1):8. doi: 10.5334/aogh.3496 (PMC8782095; doi:10.5334/aogh.3496)
Supplement: Appendix 2. — MWH Selected Qualitative Characteristics. [file agh-88-1-3496-s2.pdf]

Appendix 2: MWH Selected Qualitative Characteristics

First author, title (year)	MWH location	Establishing/ supporting agency	Cost	Admission criteria	Provisions	Facilities
Poovan, A maternity waiting home reduces obstetric catastrophes (1990)	Attat<br><br>Hospital in Ethiopia (Africa)	Faith based organizations and local villages	No direct cost to women	- Women with anticipated difficult delivery due to previous C-section, stillbirth, or ruptured uterus, or short stature<br><br>- Current pregnancy complications<br><br>- Other risk factors: grand multipara, primigravida, place of residence very far	Women required to supply their own food and firewood	- Traditional *tukul* structure with 15 beds<br><br>- Visited once a day by a nurse from the hospital
Millard, Antenatal village stay and pregnancy outcome in rural Zimbabwe (1991)	ANV located a two-minute walk from the maternity ward at Willis Pierce Hospital in Mt. Selinda, Zimbabwe	No information provided	No information provided	- Admission is open to anyone who wishes to stay<br><br>- Those with high risk pregnancies or those living far away were strongly encouraged to come (however, 83.7% of the women who stayed in the ANV had no antenatal risk factors)	No information provided	- Five rondavels that can accommodate 20-30 women at a time<br><br>- Two-minute walk from the maternity ward
van Lonkhuijzen, Use of maternity waiting home in rural Zambia (2003)	Nyanjie RCZ Hospital in rural Zambia (Africa)	No information provided	Admission fee of 1,000 Kwacha ($3) – includes cost of hospital birth (women	- In general, only high-risk women admitted, most referred by midwives from outreach clinics<br><br>- Maternal risk factors: nulliparity, grand multiparity,	Food provided by the hospital	- Accommodates 10-15 women<br><br>- Women visit the hospital's antenatal clinic once a week where they also receive health education

			admitted directly to hospital charged $6)	h/o C-section, vacuum extraction, uterine rupture, perinatal death, prolonged labor<br><br>- Antenatal risk factors: antepartum hemorrhage, multiple pregnancy, breech or transverse, pre-eclampsia<br><br>- Intrapartum risk factors: cord prolapse, hypertension, oxytocin augmentation		
Andemichael, Maternity waiting homes: A panacea for maternal/ neonatal conundrums in Eritrea (2009)	11 health facilities with MWHs in Eritrea (Africa)	Ministry of Health Eritrea	Cost of consumables provided to mothers was 750 Nakfa ($50) - no info on direct costs to women	- Reside in distant areas (>10 km from the health facility)<br><br>- Pregnant for 8 months or more	Rations of pasta, rice, oil, milk, soap, OMO, tomato paste, and cooking utensils	- 6/11 had ambulances, which often lacked tires and fuel<br><br>- 7/11 had means of communication<br><br>- all 11 had at least one health worker, nurse, or midwife trained in EmOC
Kelly, The role of a maternity waiting area (MWA) n reducing maternal mortality and stillbirths in high-risk women in rural Ethiopia (2010)	Attat<br><br>Hospital in Ethiopia (Africa)	Faith based organizations	No direct cost to women, reduction in delivery fees for those who use the MWA	- Women with risk factors from a previous pregnancy: C-section, uterine rupture, perinatal death, habitual abortion, or repaired obstetric fistula<br><br>- Women with risk factors in their current pregnancy: short primigravida, young or old primigravida, antepartum hemorrhage, pre-	Women required to obtain their own firewood and food	- Original facility was built as a tukul, until the thatched roof caught fire in 1999 and was replaced with a corrugated roof<br><br>- Hot water for showers (requested by the women)

				eclampsia, multiple pregnancy, or severe anemia		
Gaym, Maternity waiting homes in Ethiopia – three decades experience (2012)	9 MWHs in Ethiopia (Africa)	4/9 run by faith-based orgs/NGOs, 5/9 within public hospitals	Most had no admission cost, others 25-50 birr, 20-30 birr per meal	- Not always standardized or clear<br>- High risk factors: previous C-section, previous fistula repair, multiple pregnancy, malpresentations	4/9 MWHs provided food for the women, the other 5 required women to bring or buy their own food	- 7/9 had corrugated iron roofs, the other 2 were *tukuls* (mud huts with a thatched roof)<br>- Number of beds ranged from 4 to 44<br>- 3/9 had a modern kitchen<br>- 3/9 had a flush toilet
Lori, Maternity waiting homes and traditional midwives in rural Liberia (2013) AND Lori, Promoting access: the use of maternity waiting homes to achieve safe motherhood (2013)	10 rural communities in Liberia (Africa)	Community Health Development Committee, Ministry of Health and Social Welfare	Free of charge	- Available to all pregnant women<br>- Access was not dependent on referral nor the distance of a woman's home	No information provided	- TMs and family members encouraged to accompany women to the MWHs<br>- Accommodated women when extended prenatal or postnatal care required<br>- Minimum of 8 beds with mosquito netting<br>- Outdoor kitchen with cooking utensils<br>- Sheltered area for firewood<br>- Outdoor toilet facilities
Braat, Comparison of pregnancy outcomes between MWH users	Attat Hospital in Ethiopia (Africa)	No information provided	User fee dropped in 2013	- Risks relating to previous obstetric history: previous C-section, previous stillbirth<br>- Risks relating to	MWH users generally provide their own transport	- 48 beds<br>- Daily check-ups and maternal health education sessions at

and non-users at a hospitals with and without an MWH: retrospective cohort study (2018)				current pregnancy: multiple pregnancy, mal or breach, anemia, primigravida, grand multiparity, pre-eclampsia, antepartum hemorrhage<br><br>- Risk based on distance to the facility	food and firewood; hospital meals given to poor MWH users	antenatal clinic<br><br>- Asked to bring an attendant, usually their husband
Fogliati, A new use for an old tool maternity waiting homes to improve equity in rural childbirth care (2017)	Tosamaganga District-designated Hospital in Tanzania (Africa)	No information provided	Small daily fee	- Self-referred or referred by a health worker from a local facility<br><br>- No info on admission criteria<br><br>- 64.5% of MWH users and 63.0% of non-users had obstetric risk factors	No information provided	- Toilets<br><br>- Cooking facilities
Meshesha, The role of maternity waiting area in improving obstetric outcomes: comparative cross-sectional study, Jinka Zonal Hospital, Southern Regional State (2017)	Ethiopia (Africa)	No information provided	No information provided	No information provided	No information provided	No information provided
Lori, Maternity waiting homes in Liberia: Results of a countrywide multi-sector scale-up	Liberia (Africa)	Community, Ministry of Health, and NGO support	Free of charge	- Available to all pregnant women and some women postpartum<br>- No	No information provided	- Facility characteristics varied by site<br>- Family and community

(2020)				referrals or additional criteria necessary		members encouraged to accompany MWH users
Henry, Evaluating implementation effectiveness and sustainability of a maternity waiting homes intervention to improve access to safe delivery in rural Zambia: A mixed-methods protocol (2020)	Nyimba, Kalomo, Choma and Pemba Districts of Zambia (Africa)	Zambian Ministry of Health, Boston University/RTC, Africare/University of Michigan, Bill & Melinda Gates Foundation	No information provided	- No specific criteria, but women with a history of maternal or antenatal risk factors were encouraged to use the MWHs<br>- Not clearly standardized	No information provided	No information provided
Kebede, Factors influencing women's access to the maternity waiting home in rural Southwest Ethiopia: a qualitative exploration (2020)	Kaffa, Sheka, and Benchi-Maji zones of Ethiopia (Africa)	Community-owned with some governmental support	No information provided	No information provided	Food and maternity classes provided at all facilities.	- Kitchens, latrines, running water, and electricity were present at most facilities.<br>- Showers and television access were rare, with only 2 MWHs reporting these amenities.<br>- One facility was comprised of traditional

						huts while 7 were constructed with corrugated iron.
Fontanet, A Qualitative Exploration of Community Ownership of a Maternity Waiting Home Model in Rural Zambia (2020)	Chomba, Pemba, Kaloma, and Nyimba Districts of Zambia (Africa)	Community ownership	Free of charge	No information provided	Food and other material support provided by community members.	No information provided
Nigussie, Predictors of Intention to Use Maternity Waiting Home Among Pregnant Women in Bench Maji Zone, Southwest Ethiopia Using the Theory of Planned Behavior (2020)	Bench-Maji zone of Ethiopia (Africa)	Community-owned with some governmental support	Free of charge	- Rural women without easy access to a health facility (~15 days prior to estimated delivery)<br>- Women with high risk for obstetric complications	Food and water provided	No information provided
